# Neuroligin 1 modulates striatal glutamatergic neurotransmission in a pathway and NMDAR subunit-specific manner

**DOI:** 10.3389/fnsyn.2015.00011

**Published:** 2015-07-29

**Authors:** Felipe Espinosa, Zhong Xuan, Shunan Liu, Craig M. Powell

**Affiliations:** ^1^Department of Neurology and Neurotherapeutics, The University of Texas Southwestern Medical CenterDallas, TX, USA; ^2^Neuroscience Graduate Program, The University of Texas Southwestern Medical CenterDallas, TX, USA; ^3^Department of Psychiatry, The University of Texas Southwestern Medical CenterDallas, TX, USA

**Keywords:** Neuroligin 1, GluN2A, GluN2B, mEPSC, synaptic transmission, autism spectrum disorders, medium spiny neuron (MSN), NMDA

## Abstract

Together with its presynaptic partner Neurexin 1 (Nxn1), Neuroligin 1 (NL1) participates in synapse specification and synapse maintenance. We and others have shown that NL1 can also modulate glutamatergic synaptic function in the central nervous system of rodent models. These molecular/cellular changes can translate into altered animal behaviors that are thought to be analogous to symptomatology of neuropsychiatric disorders. For example, in dorsal striatum of NL1 deletion mice, we previously reported that the ratio *N*-methyl-D-aspartate receptor (NMDAR) mediated synaptic currents to α-amino-3-hydroxyl-5-methyl-4-isoxazole-propionate receptor (AMPAR) mediated synaptic currents (NMDA/AMPA) is reduced in medium spiny neuron (MSNs). Importantly, this reduction in NMDA/AMPA ratio correlated with increased repetitive grooming. The striatum is the input nucleus of the basal ganglia (BG). Classical models of this circuitry imply that there are two principal pathways that render distinct and somewhat opposite striatal outputs critical to the function of these nuclei in modulating motor behavior. Thus, we set out to better characterize the effects of NL1 deletion on direct and indirect pathways of the dorsal striatum by genetically labeling MSNs participating in the direct and indirect pathways. We demonstrate that a decrease in NMDAR-mediated currents is limited to MSNs of the direct pathway. Furthermore, the decrease in NMDAR-mediated currents is largely due to a reduction in function of NMDARs containing the GluN2A subunit. In contrast, indirect pathway MSNs in NL1 knockout (KO) mice showed a reduction in the frequency of miniature excitatory neurotransmission not observed in the direct pathway. Thus, NL1 deletion differentially affects direct and indirect pathway MSNs in dorsal striatum. These findings have potential implications for striatal function in NL1 KO mice.

## Introduction

Alterations in the postsynaptic cell-adhesion molecule Neuroligin-1 (NL1) and its trans-synaptic partner Neurexin-1 (Nxn1) are associated with neuropsychiatric disorders such as autism spectrum disorders (ASD; Szatmari et al., 2007; Kim et al., 2008a; Kirov et al., 2008, 2009; Marshall et al., 2008; Walsh et al., 2008; Yan et al., 2008; Zahir et al., 2008; Glessner et al., 2009; Gratacòs et al., 2009; Millson et al., 2012; An et al., 2014), and may also be involved in cognitive decline in the Alzheimer’s disease (Saura et al., 2011; Bie et al., 2014). These cell-adhesion molecules are critical for synapse specification and function via interactions with partners that constitute the neurotransmission machinery on both sides of the synapse (Scheiffele et al., 2000; Missler et al., 2003; Graf et al., 2004; Prange et al., 2004; Sara et al., 2005; Gerrow et al., 2006; Chubykin et al., 2007; Mukherjee et al., 2008). NL1 is specific to excitatory synapses where it promotes the retention of α-amino-3-hydroxyl-5-methyl-4-isoxazole-propionate receptors (AMPARs; Heine et al., 2008; Mondin et al., 2011) as well as the clustering of *N*-methyl-D-aspartate receptors (NMDARs) by indirect intracellular (Barrow et al., 2009) and direct extracellular interactions (Budreck et al., 2013) both early during spinogenesis and in mature synapses. Many research laboratories have identified physiological alterations in several brain regions in neuroligin models (Levinson et al., 2005; Nam and Chen, 2005; Varoqueaux et al., 2006; Chubykin et al., 2007; Futai et al., 2007; Kim et al., 2008b; Wittenmayer et al., 2009; Blundell et al., 2010; Dahlhaus et al., 2010; Jung et al., 2010; Ko et al., 2011; Mondin et al., 2011; Shipman et al., 2011; Soler-Llavina et al., 2011; Burton et al., 2012; Kwon et al., 2012; Schnell et al., 2012, 2014; Shipman and Nicoll, 2012; Hoy et al., 2013; Bie et al., 2014). Among them, the reduction in NMDA/AMPA ratio and synaptic plasticity had been repeatedly demonstrated as a result of NL1 deletion (Kim et al., 2008b; Ko et al., 2009b; Blundell et al., 2010; Dahlhaus et al., 2010; Jung et al., 2010; Shipman et al., 2011; Soler-Llavina et al., 2011; Kwon et al., 2012; Shipman and Nicoll, 2012; Hoy et al., 2013; Bie et al., 2014). Also, even though NL1 is a postsynaptic protein, altering its expression levels also has presynaptic consequences including effects on clustering of synaptic vesicles (Wittenmayer et al., 2009; Dahlhaus et al., 2010) and on the frequency of spontaneous miniature excitatory postsynaptic currents (mEPSCs; Prange et al., 2004; Nam and Chen, 2005; Chen et al., 2010; Mondin et al., 2011; Burton et al., 2012; Kwon et al., 2012; Schnell et al., 2012).

The basal ganglia (BG) are an essential component of larger parallel circuits implicated in modulation of lower-order motor control and higher-order habit formation (Villablanca and Marcus, 1975; Yin and Knowlton, 2006; Graybiel, 2008). BG circuitry is segregated in two main pathways with differential gene expression (Gerfen et al., 1990; Lobo et al., 2006) and, most importantly, with outputs of more or less opposite effects on motor and cognitive “programs” (Villablanca and Marcus, 1975; Yin and Knowlton, 2006; Graybiel, 2008). The output projections from the striatum are originated in medium spiny neurons (MSNs) of either the striatonigral (direct) pathway or the striatopallidal (indirect) pathway. Imbalances in the output of these pathways are thought to be critical in the generation of repetitive/perseverative-like behaviors in animals and humans.

Striatal synaptic alterations that correlate with repetitive behaviors have been shown in several genetic animal models (Greer and Capecchi, 2002; Welch et al., 2007; Blundell et al., 2010; Shmelkov et al., 2010; Chen et al., 2011; Peça et al., 2011; Wan et al., 2011, 2014) and could be involved in analogous symptomatology present in humans (Nicolini et al., 1996; Abelson et al., 2005; Miguel et al., 2005; Bienvenu et al., 2009). In accord, we recently demonstrated that reduced NMDA/AMPA ratio in glutamatergic projections onto striatal MSNs correlated with increased grooming in NL1 knockout (KO) mice (Blundell et al., 2010), a phenotype reversed by D-cycloserine (a NMDAR co-agonist), suggesting a relationship between a reduction in NMDAR currents and grooming.

In an effort to better define synaptic dysfunction in striatal circuits in the setting of NL1 deletion, we crossed genetic markers of direct and indirect pathway MSNs into our NL1 KO mice. These markers allow us to examine pathway-specific effects of NL1 deletion on dorsal striatum circuitry. Interestingly, we have demonstrated pathway-specific alterations in glutamatergic inputs onto direct and indirect pathway MSNs. Specifically, alterations in NMDAR-mediated currents are limited to the direct pathway while different synaptic abnormalities were demonstrated in the indirect pathway. We further refine the mechanism of reduced NMDAR function by implicating GluN2A-containing receptors. Here we replicate our previous work (Blundell et al., 2010) and extend those findings to show pathway-specific and subunit-specific alterations in NMDAR currents as well as other aspects of striatal synaptic function.

## Materials and Methods

### Mice

All experiments were performed in accord with the University of Texas Southwestern Medical Center Animal Care and Use Committee in compliance with National Institutes of Health guidelines for the care and use of experimental animals. The NL1 KO mouse was generated as described previously (Varoqueaux et al., 2006). The background of our original NL1 mutant line is a hybrid 129S6/SvEVTac X C57BL/6J cross. These mice were crossed once to mice expressing distinct fluorescent markers in the direct pathway (NL1/DR1 mice, td-Tomato driven by the dopamine-1 receptor promoter; Shuen et al., 2008) and in the indirect pathway (NL1/DR2 mice, enhanced green fluorescent protein driven by the dopamine-2 receptor promoter; Gong et al., 2003). In an effort to preserve the original hybrid background, we then backcrossed each of these “labeled” mice for five generations into the original hybrid NL1 mutant background. In order to obtain mice in which both D1 and D2 MSNs were labeled, we crossed heterozygous NL1 mice with the fluorescent markers to one another for three generations. NL1 heterozygous F3 mice expressing markers in both pathways were then used as breeders to generate WT and NL1 KO individuals for experiments as well as NL1 heterozygous mice to replace breeders. These crosses created the added benefit of uniform genetic background across all experiments.

Recently the use of bacterial artificial chromosome (BAC) transgenic mice expressing EGFP driven by the DR2 promoter had been called into question because, when comparing to wild-type (WT) mice, an increased expression of the D2 receptor as well as a mild hyperactive phenotype were found in these mice but not in BAC transgenic mice expressing either EGFP or td-Tomato in the direct pathway (Ade et al., 2011; Kramer et al., 2011). Later reports from several groups suggest that strain-specific, high-sensitivity may have been the culprit for those findings (Chan et al., 2012; Nelson et al., 2012). In the present study, comparisons are made between WT and NL1 KO mice that both express either D1 or D2 labeled BAC transgenes. Thus, any effects of these transgenes are equivalent in both WT and NL1 KO groups. Furthermore, our use of hybrid genetic backgrounds may obviate this potential confound (Gertler et al., 2008; Grueter et al., 2010; Lobo et al., 2010; Chan et al., 2012; Nelson et al., 2012).

### Genotyping

Genotyping was performed by PCR of genomic DNA extracted from tails. For NL1 genotyping the following primers were used: MB0606 (WT-specific), 5′-CGA GAG TCA GGT AAA TTG AAC ACC AC-3′; T676 (KO-specific), 5′-GAG CGC GCG CGG CGG AGT TGT TGA C-3′; and T1660 (common), 5′-GTG AGC TGA ATC TTA TGG TTA GAT GGG-3′. Importantly, given that amplification of the mutant PCR fragment was significantly more efficient than the amplification of the WT PCR fragment, we used a 3:1–4:1 ratio of WT-specific primer vs. the KO-specific primer. PCR products were run in a 1.6–1.8% agarose gel. WT and KO PCR product sizes were: ~550 bp and ~415 bp, respectively. The PCR protocol was: 1 step at 98°C for 3 min; 30 cycles of 98°C for 15 s, 65°C for 30 s and 72°C for 20 s; a final step at 72°C for 5 min. Genotyping for DR1-tdTomato transgene or for DR2-EGFP transgene, was done using same procedures as in Ade et al. (2011).

### Striatal Neurotransmission

Horizontal-oblique slices were prepared from NL1/D1 and D2 mouse brains, and acute slice striatal recordings were performed as previously described (Ding et al., 2008; Smeal et al., 2008; Blundell et al., 2010). Briefly, 2–3 week old WT and KO mice were anesthetized with ketamine/xylazine (20%/10%, respectively) in 0.85% (w/v) saline cocktail, perfused intracardially with 10–20 ml of artificial cerebrospinal fluid (aCSF), and decapitated. The brain was quickly isolated and chilled in dissecting solution. The dissecting solution contained the following (in mM): 75 sucrose, 81 NaCl, 2.5 KCl, 1.0 NaH_2_PO_4_, 0.1 CaCl_2_, 4.9 MgCl_2_, 26.2 NaHCO_3_, and 1.5 glucose (300–305 mOsm). Slices 360 μm thick were cut using a model 300 Vibratome. Slices were incubated in the bathing solution at 32 ± 1°C for 30–40 min. Slices were then stored at room temperature until transferred to a submersion-type recording chamber after at least 80 min of recovery following slicing. The bathing solution (regular aCSF) contained (in mM): 124 NaCl, 3 KCl, 1.25 NaH_2_PO_4_, 2 CaCl_2_, 1 MgSO_4_, 26 NaHCO_3_, and 1 glucose (saturated with 95% O_2_/5% CO_2_). Whole-cell patch-clamp recordings from striatal tdTomato- or from eGFP-expressing MSNs were performed using micropipettes with a resistance of 3–5 MΩ made from 1.1/1.5 mm borosilicate glass (Sutter Instruments). Recording pipettes were filled with the following solution (in mM): 117 Cs-methanesulphonate, 2.8 NaCl, 5 TEA-Cl, 2 QX-314 [*N*-(2,6-dimethylphenylcarbamoylmethyl)triethylammonium chloride], 0.4 EGTA, 2 ATP-Mg, 0.25 GTP-Mg, 20 HEPES-CsOH (pH 7.2–7.4, 275–285 mOsm). A junction potential of 12 mV between the internal and the external solution was calculated theoretically (using the corresponding function in Clampfit), corroborated empirically, and used to correct voltages online. Access resistance was frequently checked to be <30 MΩ and stable (less than 20% variability). Protocols were designed to study input/output curves, NMDA/AMPA ratio, miniature EPSCs and, paired pulse ratio (PPR; see below). At all times, at least five breeding pairs were kept as resource for experimental mice. For each genotype/striatal pathway/protocol, useful data sets were obtained from six or more mice from three or more litters, where 1–3 cells per slice/pathway and 1–5 cells per mouse/pathway were recorded. Results from a total of 185 WT (91 DR1 and 94 DR2) cells and a total of 182 KO (89 DR1 and 93 DR2) cells are reported. Baseline parameters in regular aCSF and with the Cs^+^-based internal solution used here did not differ among the groups including access resistance [(DR1 cells: 17.0 ± 0.70 MΩ, WT; 18.6 ± 0.70 MΩ, NL1 KO, *P* = 0.111; DR2 cells: 19.0 ± 0.79 MΩ, WT; 17.8. ± 0.77 MΩ, NL1 KO, *P* = 0.361)], cell membrane resistance [(DR1 cells: 197 ± 10.3 MΩ, WT; 229 ± 13.2 MΩ,NL1 KO, *P* = 0.114; DR2 cells: 312 ± 23.3 MΩ, WT; 326 ± 23.0 MΩ,NL1 KO, *P* = 0.276)], and cell capacitance [(DR1 cells: 139 ± 4.8 PF, WT; 147 ± 4.35 PF, NL1 KO, *P* = 0.231; DR2 cells: 103 ± 3.8 PF, WT; 101.2 ± 3.4 PF, NL1 KO, *P* = 0.361)].

**Figure 1:**
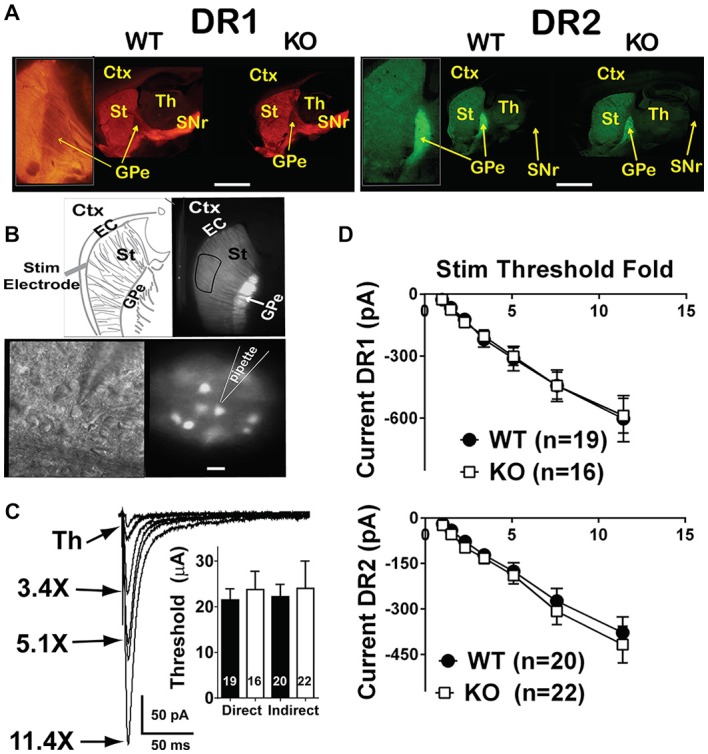
**Input/output curves of glutamatergic projections onto direct and indirect pathway MSNs are unchanged. (A)** Fluorescent microscope low power images of WT and NL1 KO mice expressing the fluorescent markers td-Tomato (driven by the DR1 promoter, direct pathway, left panels) or EGFP (driven by the DR2 promoter, indirect pathway, right panels). Insets show projections of DR1 MSNs to SNr passing through the GPe (left inset) or a strongly glowing GPe due to EGFP-expressing DR2 terminals from the striatum (right inset). Inset on DR1 WT was taken from a different image that better highlighted the DR1 medium spiny neuron (MSN) projections. Bar = 2 mm (0.8 mm for insets). **(B)** Upper panels, low power fluorescence image of a DR2 mouse showing the target site for recording MSNs (right) and schematic showing the placement site for the stimulation electrode (left). Bottom panels, high power infrared differential interference contrast and DOT optics (right) or DR2-EGFP fluorescence (left) images showing the approach of a recording pipette to an EFGP-expressing MSN. Bar = 25 μm for bottom images and 500 μm for top images. **(C)** Traces of evoked EPSCs (eEPSCs) elicited upon current injections onto the EC, and recorded in whole-cell configuration in striatal MSNs. The intensity of the stimulation, expressed as a multiple of the threshold, is connected to the corresponding trace by an arrow. Inset. For I/O recordings, threshold stimulation was required to be below 60 μA. Under this condition, stimulation threshold was similar between WT and NL1 KO mice. **(D)** I/O curves for the direct (left) or the indirect (right) pathways are similar between genotypes. Labels: Ctx, cortex; EC, external capsule; St, striatum; Th, thalamus; GPe, globus pallidus externus and SNr, substantia nigra pars reticulata.

EPSCs were evoked (eEPSCs) by stimulating the brain at the boundary of the corpus callosum and the dorsolateral striatum. Stimuli were elicited for 0.2 ms using bipolar electrodes and a model A365 battery-driven stimulus isolator (WPI). For input/output (I/O) experiments, the lowest stimulation intensity to elicit events in at least three out of five stimulations was considered the threshold for the experiment. The intensity of the stimulus was then scaled 1.5× the previous intensity and in some experiments, up to 25.6× threshold (labeled as “Threshold Fold” in Figure 1C). I/O linearity was usually lost in stimulation intensities around 17.4× threshold; therefore, I/O analysis was done on stimulation intensities that ranged from threshold to 11.4× threshold. Evoked NMDA/AMPA ratios were determined using standard, published methods (Myme et al., 2003). In the presence of picrotoxin, AMPAR currents were measured at the peak and at a voltage of −80 mV, at which most NMDAR currents are blocked by Mg^2+^. Stimulation intensities were adjusted so the peak amplitude of the AMPA current was maintained within the 200–600 pA (mostly within 300–500 pA) range. NBQX (10 μM), an AMPA receptor antagonist, was added to the bath after recording AMPAR currents. In the same cell, NMDAR currents were measured in a 2 ms window 48 ms after stimulus onset at a voltage of +40 mV. In a subset of these experiments Ro 25–6891 (0.5–1 μM), an antagonist of GluN2B-containing NMDARs, was added to the bath. To determine the current contribution of GluN2B-containing NMDARs vs. the current contributed by putative GluN2A-containing NMDARs, the remaining current after application of Ro 25–6891 (the putative GluN2A component) was subtracted off-line from the total NMDAR current to obtain the GluN2B-component. PPR was done at two inter-event intervals (IEIs) that corresponded to the maximal facilitation (40 ms) or the maximal depression (400 ms; Akopian and Walsh, 2007; Ding et al., 2008). Stimulation intensities were adjusted so the peak amplitude of the first pulse was maintained within the 150–300 pA range. Even though stimulus intensities were intended to induce a homogenous conditioning stimulus across groups, the resulting peak current amplitudes for P1 differed among them (not shown). Therefore, to cancel this artificial difference, all events within a group were normalized to the average current amplitude for P1. These normalized data was then used for statistical analysis to compare P1 vs. P2 between genotypes and genders and presented in Table 2 as normalized event amplitude 1 of short inter-pulse interval (NE1S) vs. NE2S or as normalized event amplitude 1 of long inter-pulse interval (NE1L) vs. NE2L. For recording of mEPSCs, the bathing solution was supplemented with Tetrodotoxin (0.75–1 μM) and Picrotoxin (50–100 μM). After breaking in, the internal solution was dialyzed for at least 8 min, then cells were held at −70 mV and spontaneous mEPSC transmission was recorded for 2–5 min or until at least 200 events were obtained.

Recordings were obtained using the 700B Multiclamp amplifier (Molecular Devices, Sunnyvale, CA, USA), and neurons were visualized using a Carl Zeiss Axioexaminer D1 microscope equipped with infrared differential interference contrast, DOT optics, a CCD camera, and epifluorescence. The fluorescence filters used were (excitation, beam splitter, emission): set 38 (BP 470/40, FT 495, BP 525/50) for EGFP expressing DR2 cells and, set 43 (BP 545/25, FT 570, BP 605/70) for td-Tomato expressing DR1 cells (Carl Zeiss Microimaging Inc., Thornwood, NY, USA). Responses were digitized at 10 kHz and filtered at 2 kHz. Data were analyzed offline using pClamp (Molecular Devices, Sunyvale, CA, USA), Minianalysis (Synaptosoft, Fort Lee, NJ, USA), and Microsoft Excel, Redmond, WA, USA.

### Statistical Analysis

All statistical analyses were conducted using Statistica software (version 5.5; Statsof, Tulsa, OK, USA) or Prism software (version 6.02; GraphPad, La Jolla, CA, USA). Statistica was used either for two-way ANOVAs or for three-way repeated measures ANOVA (rmANOVA). Sex and genotype were the two factors in two-way ANOVAs. In addition, for the three-way ANOVA, either the stimulus threshold fold (STF; for I/O curves), or the pulse number (for PPR) were used. If a main effect of sex was found, the Tukey Honest Significant Difference with unequal N (the Spjotvoll-Stoline) *post hoc* test was applied. Both male and female mice were used for the experiments. For the purpose of these studies, where the effects of constitutively deleting the NL1 protein are central to our investigations, it was required that the Spjotvoll-Stoline test rendered a significant difference when comparing genders within the same genotype. Otherwise, any statistically significant “main effect of sex” obtained in the analysis was considered either spurious or of little value to understand the importance of NL1-deletion in striatal pathophysiology. Importantly, not a single parameter fulfilled the above requirement (see Tables 1, 2), therefore, genders were pooled throughout this work. When appropriate, Graphpad Prism software was used for linear regression analysis where slope = *r*^2^ and probability are reported. For ease of flow of data presentation, only rmANOVAs corresponding to the analysis of I/O curves are presented as part of the main text. For the rest of the data, average ± SEM are presented, and *P* values correspond to those from the ANOVA analysis. *P* values between 0.1005 and 0.0504 were considered of potential interest to readers and were included in the figures. For further reference, a complete display of the statistical results for ANOVAs and rmANOVAs is presented in Tables 1, 2.

**Table 1:** **Analysis of variance for electrophysiological studies**.

Parameter	Comparison	Results
**I/O Direct; WT *n* = 19, KO *n* = 16**
	Sex and Genotype and Stimulus Threshold Fold (STF)	3-way rmANOVA; Main effect of sex, *F*_(1,30)_ < 0.21, *p* < 0.652; main effect of genotype, *F*_(1,30)_ < 0.01, *p* < 0.937; **main effect of stimulus threshold fold (STF), *F*_(6,180)_ < 60.11, *p* < 0.0001**; sex × genotype interaction, *F*_(1,30)_ < 3.46, *p* < 0.073; sex × STF interaction, *F*_(6,180)_ < 1.24, *p* < 0.289; genotype × STF interaction, *F*_(6,180)_ < 0.07, *p* < 0.999; **sex × genotype × STF interaction; *F*_(6,180)_ < 2.24, *p* < 0.041**
**I/O Indirect; WT *n* = 20, KO *n* = 22**
	Sex and Genotype and STF	3-way rmANOVA; Main effect of sex, *F*_(1,33)_ < 0.63, *p* < 0.433; main effect of genotype, *F*_(1,33)_ < 0.09, *p* < 0.768; **main effect of stimulus threshold fold STF, *F*_(6,198)_ < 43.24, *p* < 0.0001**; sex × genotype interaction, *F*_(1,33)_ < 0.41, *p* < 0.529; sex × STF interaction, *F*_(6,189)_ < 0.34, *p* < 0.917; genotype × STF interaction, *F*_(6,189)_ < 0.05, *p* < 0.999; sex × genotype × STF interaction; *F*_(6,189)_ < 0.66, *p* < 0.686
**NMDAR Current; Direct WT *n* = 31, KO *n* = 33**
NMDAR current	Sex and Genotype	2-way ANOVA; Main effect of sex, *F*_(1,61)_ < 0.07, *p* < 0.799; **main effect of genotype, *F*_(1,61)_ < 11.86, *p* < 0.001; sex × genotype interaction, *F*_(1,61)_ < 4.05, *p* < 0.049;**
NMDA/AMPA ratio	Sex and Genotype	2-way ANOVA; Main effect of sex, *F*_(1,61)_ < 0.004, *p* < 0.948; **main effect of genotype, *F*_(1,61)_ < 8.12, *p* < 0.006; sex × genotype interaction, *F*_(1,61)_ < 6.54.05, *p* < 0.013**
**NMDAR Current; Indirect WT *n* = 29, *n* = 31**
NMDAR current	Sex and Genotype	2-way ANOVA; Main effect of sex, *F*_(1,59)_ < 1.48, *p* < 0.2293; main effect of genotype, *F*_(1,59)_ < 1.18, *p* < 0.28; sex × genotype interaction, *F*_(1,59)_ < 1.57, *p* < 0.216
NMDA/AMPA ratio	Sex and Genotype	2-way ANOVA; Main effect of sex, *F*_(1,59)_ < 0.62, *p* < 0.434; main effect of genotype, *F*_(1,59)_ < 1.96, *p* < 0.167; sex × genotype interaction, *F*_(1,61)_ < 1.34, *p* < 0.258
**NMDAR Charge transfer (Q); Direct WT *n* = 20, KO *n* = 21**
Total NMDAR Q	Sex and Genotype	2-way ANOVA; Main effect of sex, *F*_(1,39)_ < 2.18, *p* < 0.148; **main effect of genotype, *F*_(1,59)_ < 7.78, *p* < 0.008**; sex × genotype interaction, *F*_(1,59)_ < 0.64, *p* < 0.429
GluN2A Q	Sex and Genotype	**2-way ANOVA; Main effect of sex, *F*_(1,39)_ < 5.0, *p* < 0.031; main effect of genotype, *F*_(1,39)_ < 7.80, *p* < 0.008;** sex × genotype interaction, *F*_(1,39)_ < 0.13, *p* < 0.726
GluN2B Q	Sex and Genotype	2-way ANOVA; Main effect of sex, *F*_(1,39)_ < 0.01, *p* < 0.933; main effect of genotype, *F*_(1,39)_ < 3.04, *p* < 0.089; sex × genotype interaction, *F*_(1,39)_ < 1.20, *p* < 0.281
**NMDAR Q; Indirect WT *n* = 21, KO *n* = 23**
Total NMDAR Q	Sex and Genotype	2-way ANOVA; Main effect of sex, *F*_(1,39)_ < 0.17, *p* < 0.687; main effect of genotype, *F*_(1,59)_ < 2.51, *p* < 0.121; sex × genotype interaction, *F*_(1,59)_ < 0.90, *p* < 0.348
GluN2A Q	Sex and Genotype	2-way ANOVA; Main effect of sex, *F*_(1,39)_ < 0.53, *p* < 0.473; main effect of genotype, *F*_(1,39)_ < 1.13, *p* < 0.295; sex × genotype interaction, *F*_(1,39)_ < 0.34, *p* < 0.561
GluN2B Q	Sex and Genotype	2-way ANOVA; Main effect of sex, *F*_(1,39)_ < 0.01, *p* < 0.931; main effect of genotype, *F*_(1,39)_ < 2.52, *p* < 0.120; **sex × genotype interaction, *F*_(1,39)_ < 5.23, *p* < 0.028**
**Miniature neurotransmission direct; WT *n* = 18, KO *n* = 18**
Amplitude	Sex and Genotype	2-way ANOVA; Main effect of sex, *F*_(1,32)_ < 0.003, *p* < 0.954; main effect of genotype, *F*_(1,32)_ < 1.32, *p* < 0.260; sex × genotype interaction, *F*_(1,32)_ < 0.18, *p* < 0.673
Frequency	Sex and Genotype	2-way ANOVA; Main effect of sex, *F*_(1,32)_ < 3.50, *p* < 0.070; main effect of genotype, *F*_(1,32)_ < 0.15, *p* < 0.697; sex × genotype interaction, *F*_(1,32)_ < 1.82, *p* < 0.186
**Miniature neurotransmission indirect; WT *n* = 20, KO *n* = 20**
Amplitude	Sex and Genotype	2-way ANOVA; Main effect of sex, *F*_(1,32)_ < 3.96, *p* < 0.054; main effect of genotype, *F*_(1,32)_ < 3.04, *p* < 0.090; sex × genotype interaction, *F*_(1,32)_ *F*_(1,36)_ < 0.05, *p* < 0.831
Frequency	Sex and Genotype	2-way ANOVA; Main effect of sex, *F*_(1,32)_ < 0.187, *p* < 0.668; **main effect of genotype, *F*_(1,32)_ < 5.67, *p* < 0.023**; sex × genotype interaction, *F*_(1,32)_ *F*_(1,36)_ < 0.03, *p* < 0.860
**PPR Direct; WT *n* = 20, KO *n* = 20**
Norm P1 vs. Norm P2 (IEI 400 ms)	Sex and Genotype and pulse # (P)	3-way ANOVA; Main effect of sex, *F*_(1,36)_ < 1.87, *p* < 0.180; main effect of genotype, *F*_(1,36)_ < 0.29, *p* < 0.593; **main effect of P, *F*_(6,180)_ < 251.4, *p* < 0.0001**; sex × genotype interaction, *F*_(1,36)_ < 0.299, *p* < 0588; **sex × P interaction, *F*_(1,36)_ < 5.79, *p* < 0.021**; genotype × P interaction, *F*_(2,36)_ < 0.29, *p* < 0.594; sex × genotype × P interaction; *F*_(1,36)_ < 0.1, *p* < 0.752
Norm P1 vs. Norm P2 (IEI 40 ms)	Sex and Genotype and P	3-way ANOVA; Main effect of sex, *F*_(1,36)_ < 0.03, *p* < 0.876; main effect of genotype, *F*_(1,36)_ < 1.01, *p* < 0.321; **main effect of P**, *F*_(6,180)_ < 9.55, *p* < 0.004; sex × genotype interaction, *F*_(1,36)_ < 0.05, *p* < 0.889; sex × P interaction, *F*_(1,36)_ < 0.10, *p* < 0.756; genotype × P interaction, *F*_(1,36)_ < 3.48, *p* < 0.070; sex × genotype × P interaction; *F*_(1,36)_ < 0.08, *p* < 0.780
P1 P2 (IEI 400 ms)	Sex and Genotype	2-way ANOVA; **Main effect of sex, *F*_(1,39)_ < 6.93, *p* 0.012**; main effect of genotype, *F*_(1,39)_ < 0.181, *p* < 0.187; sex × genotype interaction, *F*_(1,39)_ < 0.05, *p* < 0.824
P1 P2 (IEI 40 ms)	Sex and genotype	2-way ANOVA; Main effect of sex, *F*_(1,39)_ < 0.001, *p* < 0.973; main effect of genotype, *F*_(1,39)_ < 1.01, *p* < 0.321; sex × genotype interaction, *F*_(1,32)_ < 0.17, *p* < 0.682
**PPR Indirect; WT *n* = 19, KO *n* = 21**	
Norm P1 vs. Norm P2 (IEI 400 ms)	Sex and Genotype and pulse # (P)	3-way ANOVA; Main effect of sex, *F*_(1,36)_ < 0.04, *p* < 0.834; main effect of genotype, *F*_(1,36)_ < 0.02, *p* < 0.904; **main effect of P, *F*_(6,180)_ < 102.73, *p* < 0.0001**; sex × genotype interaction, *F*_(1,36)_ < 0.01, *p* < 0.929; sex × P interaction, *F*_(1,36)_ < 0.72, *p* < 0.400; genotype × P interaction, *F*_(2,36)_ < 0.20, *p* < 0.656; sex × genotype × P interaction; *F*_(1,36)_ < 0.14, *p* < 0.714
Norm P1 vs. Norm P2 (IEI 40 ms)	Sex and Genotype and P	3-way ANOVA; Main effect of sex, *F*_(1,30)_ < 0.12, *p* < 0.728; main effect of genotype, *F*_(1,30)_ < 0.13, *p* < 0.717; **main effect of P, *F*_(6,180)_ < 25.46, *p* < 0.001**; sex × genotype interaction, *F*_(1,36)_ < 0.51, *p* < 0.479; sex × P interaction, *F*_(1,36)_ 2.36, *p* < 0.134; genotype × P interaction, *F*_(2,36)_ < 2.42, *p* < 0.129; sex × genotype × P interaction; *F*_(1,36)_ < 9.13, *p* < 0.004
P1 P2 (IEI 400 ms)	Sex and Genotype	2-way ANOVA; Main effect of sex, *F*_(1,39)_ < 0.50, *p* < 0.485; main effect of genotype, *F*_(1,39)_ < 1.05, *p* < 0.313; sex × genotype interaction, *F*_(1,39)_ < 0.04, *p* < 0.547
P1 P2 (IEI 40 ms)	Sex and genotype	2-way ANOVA; Main effect of sex, *F*_(1,39)_ < 3.23, *p* < 0.081; main effect of genotype, *F*_(1,39)_ < 2.09, *p* < 0.157; **sex × genotype interaction, *F*_(1,39)_ < 6.25, *p* < 0.017**

*Either 2-way ANOVAs (for glutamatergic currents and charge transfer, and for NMDA/AMPA ratios and P2/P1 ratios between genotypes) or 3-way ANOVAs with repeated measures (for I/O curves or for comparison of P1 vs. P2 amplitude within genotypes) were used. Between-subjects factors are generally sex and genotype, with repeated measures (pulse number either in the I/O curves or in PPR). F(x, y), F ratio statistic is used to determine whether the variances in two independent samples are equal; x, y are degrees of freedom. Degrees of freedom is a measure of the number of independent pieces of information on which the precision of a parameter estimate is based. x, Number of groups −1; y, number of animals per group −1, multiplied by the number of groups*.

**Table 2:** **Probability summary of main effect of sex on NL1KO physiology**.

	DR1	DR1 *post hoc* Spjotvoll-Stoline test		DR2
*Parameter*	*All*	*WT*	*KO*	*All*
		*M vs. F*	*M vs. F*
I/O	*0.652*	*−*	*−*	*0.433*
NMDAR current	*0.799*	*−*	*−*	*0.229*
NMDA/AMPA ratio	*0.948*	*−*	*−*	*0.434*
NMDA Q	*0.148*	*−*	*−*	*0.687*
GluN2A Q	**0.031**	*0.612*	*0.297*	*0.473*
GluN2B Q	*0.933*	*−*	*−*	*0.931*
Mini Amp	*0.954*	*−*	*−*	*0.054*
Mini Freq	*0.070*	*−*	*−*	*0.668*
PPR: NP1S vs. NP2S	*0.875*	*−*	*−*	*0.727*
PPR: P1S/P2S	*0.973*	*−*	*−*	*0.081*
PPR: NP1L vs. NP2L	*0.180*	*−*	*−*	*0.834*
PPR: P1L/P2L	**0.012**	*0.381*	*0.198*	*0.313*

*The Spjotvoll-Stoline post hoc test for unequal n_s_ was performed on parameters that showed a main effect of sex in the ANOVAs. However, when genders within a genotyped were compared, no significant difference was found, therefore, genders were pooled. NPS, Normalized Event Amplitude, Short Interpulse Interval (40 ms). NPL, Normalized Event Amplitude, Long Interpulse Interval (400 ms)*.

## Results

### Strength of Glutamatergic Inputs onto MSNs of the Direct and of the Indirect Pathways

Mice constitutively deficient in NL1 show alterations in glutamatergic synaptic function (Chubykin et al., 2007; Blundell et al., 2010). These mice, however, demonstrate increased repetitive grooming behaviors that are potentially mediated by the striatum along with a decrease in NMDA/AMPA ratio at excitatory inputs onto striatal MSNs (MSNs; Blundell et al., 2010). This suggests either a decrease in NMDAR-mediated current, an increase in AMPAR-mediated current or some combination of the two. Measuring NMDA/AMPA ratio is also not useful for determining baseline synaptic strength. Thus, to compare synapse strength between genotypes, we examined input/output (I/O) curves of evoked excitatory synaptic transmission onto both DR1 (direct pathway) and DR2 (indirect pathway) MSNs of NL1 KO mice compared to WT littermate controls (Figure 1A). Synaptic currents were evoked by stimulation in the nearby corpus callosum. Recordings were done at a membrane potential of −80 mV, where the slowly activating NMDA receptors are blocked by extracellular Mg^2+^ ions. Due to this and because current amplitudes were determined at the peak of the eEPSCs, this experimental protocol mainly determines the strength of AMPA receptor signaling. Typically, stimulation thresholds to elicit a recognizable eEPSC response in three out of five stimuli were between the 15–30 μA. To reduce the likelihood of evoking polysynaptic events due to stimulation of clusters of fibers distant to the stimulating electrode, experiments with thresholds above 60 μA were not used in I/O analysis. Using these parameters, maximum stimulation intensities never exceeded 700 μA. No main effect of gender or of genotype was found in either pathway (Figures 1B–D; I/O three way rmANOVA. DR1 MSNs: main effect of sex, *F*_(1,30)_ = 0.21, *P* = 0.652; main effect of genotype, *F*_(1,30)_ = 0.01, *P* = 0.937; main effect of STF, *F*_(6,180)_ = 60.11, *P* < 0.0001; sex × genotype interaction, *F*_(1,30)_ = 3.46, *P* = 0.073; sex × STF interaction, *F*_(6,180)_ = 1.24, *P* = 0.289; genotype × STF interaction, *F*_(6,180)_ = 0.07, *p* = 0.999; sex × genotype × STF interaction; *F*_(6,180)_ = 2.24, *P* = 0.041; DR2 MSNs: main effect of sex, *F*_(1,33)_ = 0.63, *P* = 0.433; main effect of genotype, *F*_(1,33)_ 0.09, *P* = 0.768; main effect of STF, *F*_(6,198)_ = 43.24, *P* < 0.0001; sex × genotype interaction, *F*_(1,33)_ = 0.41, *P* = 0.529; sex × STF interaction, *F*_(6,189)_ = 0.34, *P* = 0.917; genotype × STF interaction, *F*_(6,189)_ = 0.05, *P* = 0.999; sex × genotype × STF interaction; *F*_(6,189)_ = 0.66, *P* = 0.686). In line with previous reports in other brain regions on NL1 KO mice (Chubykin et al., 2007; Jung et al., 2010; Soler-Llavina et al., 2011), these experiments suggest that baseline, largely AMPAR-mediated glutamatergic evoked synaptic responses are not altered in the striatum of NL1 KO mice.

### NMDA/AMPA Ratio is Selectively Reduced in the Direct Pathway

Both intracellular and extracellular domains of NL1 are thought to modulate clustering of NMDARs at the synapse (Barrow et al., 2009; Budreck et al., 2013). This is in line with the strong correlation between NMDA/AMPA ratio (recorded in cells in culture as well as in acute slices) and NL1 expression levels manipulated by diverse strategies. In general, overexpression increases the NMDA/AMPA ratio whereas knockdown or knockout strategies result in decreases in NMDA/AMPA ratio (Chubykin et al., 2007; Futai et al., 2007; Kim et al., 2008b; Jung et al., 2010; Soler-Llavina et al., 2011; Kwon et al., 2012; Budreck et al., 2013). Because NL1 KO mice exhibit reduced NMDA/AMPA ratio in MSNs in the striatum (Blundell et al., 2010), we further examined NMDA/AMPA ratio specifically in DR1 or in DR2 MSNs of WT and NL1 KO mice.

**Figure 2:**
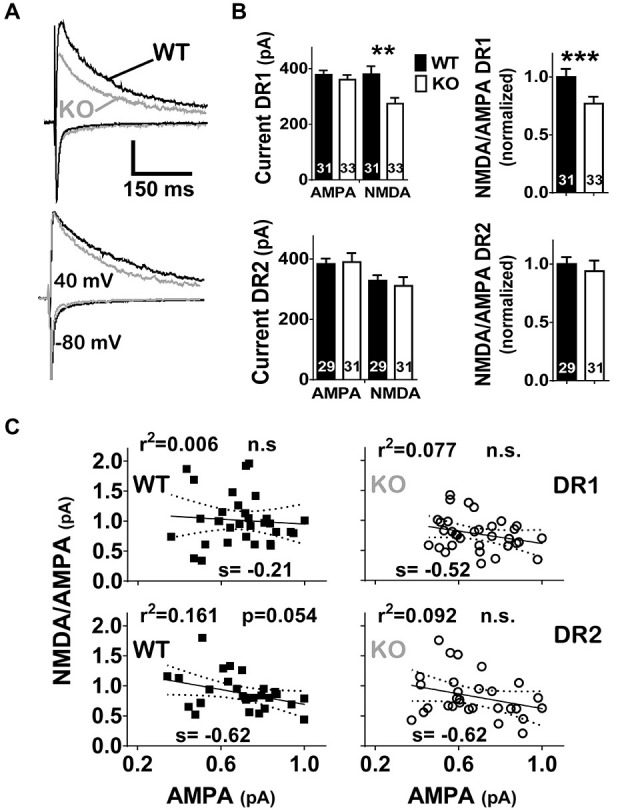
**NL1 KO mice show a DR1 specific reduction in NMDAR neurotransmission. (A)** eEPSCs were elicited as in Figure 1 but AMPAR current peak was required to be in the range of 200–600 pA. Traces of WT (black) or KO (gray) mice were superimposed. The amplitude of AMPAR current for the trace corresponding to the DR1-WT cell was 522 pA. The amplitude of the AMPAR current for all other cells in this figure were scaled to that of DR1-WT and all NMDAR currents were proportionally scaled. Actual amplitudes for scaled-AMPAR currents in this figure were: 437 pA, 378 pA and 404 pA, for DR1-NL1 KO, DR2-NL1 WT and DR2-NL1 KO cells, respectively. Vertical bar = 250 pA. **(B)** Bar graphs of current amplitudes (left) for AMPARs and NMDARs or for the NMDA/AMPA ratio (right). NL1 KO mice showed a deficit in NMDAR currents, as well as a reduction in the NMDA/AMPA ratio, but only in the direct pathway. **p < 0.01, ***p < 0.001. **(C)** Regression analysis of normalized AMPAR currents vs. the NMDA/AMPA ratio. AMPAR currents of NL1 KO mice were normalized to the maximal peak current for their WT control group within the direct or indirect pathway. The NMDA/AMPA ratio showed no significant correlation to the normalized amplitude of the AMPAR current.

To limit stimulation to local fibers, stimulation intensities were adjusted to elicit fast AMPA eEPSCs with amplitudes between 200–600 pA (mostly between 300–500 pA). When compared to WT littermates, NL1 KO mice showed a significant decrease in NMDAR current amplitude (WT, 380 ± 28.2 pA, *n* = 32, vs. KO, 274 ± 21.2 pA, *n* = 33, *P* = 0.001) and in NMDA/AMPA ratio (WT, 1.01 ± 0.07, *n* = 32, vs. KO, 0.78 ± 0.06, *n* = 33, *P* = 0.006) on direct pathway MSNs. In contrast, neither a change in NMDAR current amplitude (WT, 328 ± 19.5 pA, *n* = 28, vs. KO, 311 ± 28.7 pA, *n* = 29, *P* = 0.283), nor in NMDA/AMPA ratio (WT, 0.88 ± 0.06, *n* = 28, vs. KO, 0.83 ± 0.08, *n* = 29, *P* = 0.167), was observed on DR2 MSNs (Figures 2A,B). Regression analysis further confirms no impact of the amplitude of the AMPA eEPSC on the NMDA/AMPA ratio under any condition (Figure 2C; Linear Regression. DR1 WT; slope = −0.21 ± 0.47, *r*^2^ = 0.006, *F*_(1,30)_ = 0.184, *p* = 0.671; DR1 KO; slope = −0.52 ± 0.32, *r*^2^ = 0.077, *F*_(1,31)_ = 2.57; DR2 WT; slope = −0.62 ± 0.31, *r*^2^ = 0.136, *F*_(1,26)_ = 0.4.09, *p* = 0.535; DR2 KO; slope = −0.62 ± 0.37, *r*^2^ = 0.092, *F*_(1,27)_ = 2.73, *P* = 0.110).

### The Reduction in NMDAR Currents in the Direct Pathway is Mainly Driven by Decreased GluN2A Currents

NMDARs are heterotetramers composed of two GluN1 subunits and two GluN2 or GluN3 subunits (Kutsuwada et al., 1992; Das et al., 1998; Laube et al., 1998). There are four isoforms for GluN2 (GluN2A-D) that have distinctive biophysical and physiological properties (Köhr et al., 2003; Massey et al., 2004; Erreger et al., 2005; Kim et al., 2005; Foster et al., 2010; Wyllie et al., 2013). Only Glun2A and GluN2B are detected at significant levels in MSNs in the striatum (Landwehrmeyer et al., 1995; Kuppenbender et al., 2000; Dunah and Standaert, 2003). Recent evidence suggests that these subunits have a divergent influence on striatal physiology and on correlated behaviors (Fantin et al., 2007; Sarre et al., 2008; Schotanus and Chergui, 2008; Mabrouk et al., 2013).

**Figure 3:**
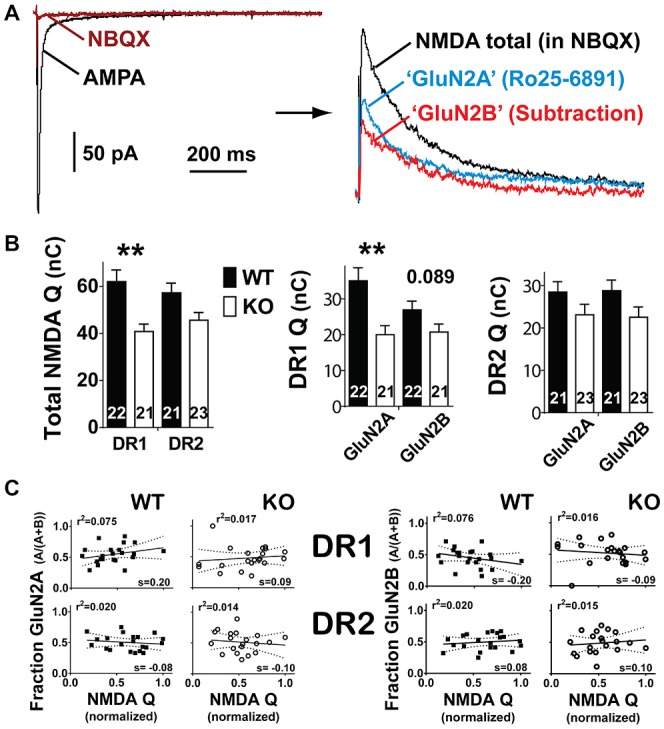
**Reduction in NMDAR currents is mainly due to a decrease in GluN2A currents. (A)** AMPA receptor currents were blocked with the antagonist 5 μM NBQX (left). Afterwards, baseline NMDAR currents were recorded at +40mV followed by the application of 0.5–1 μM Ro25–6891 (a GluN2B antagonist, right). The remaining current (blue trace) is considered to be driven by NMDARs containing GluN2A subunits. To estimate the contribution of GluN2B subunits to the total NMDAR current, the GluN2A component was subtracted from the total. The difference (red trace) was considered to be driven by GluN2B. **(B)** Left panel. Integration of total NMDAR current confirmed the selective reduction in this conductance in the direct pathway. Middle and right panels. In the direct pathway, GluN2A was significantly reduced whereas GluN2B shows a trend towards reduction. Instead, in the indirect pathway neither conductance showed significant changes. **p < 0.01. **(C)** Regression analysis of NMDAR currents (normalized) vs. the fraction of GluN2A (left) or the fraction GluN2B (right). NMDAR currents were normalized to the maximal peak current for their WT control group within the direct and indirect pathways. No significant correlation was found between the amplitude of the normalized NMDAR current and the fraction contributed by GluN2A or GluN2B currents in either pathway.

To further unravel the possible physiological impact of the reduced NMDAR currents in the striatum of NL1 KO mice, we determined if this alteration is selective for GluN2A- vs. GluN2B-containing NMDARs. After recording baseline NMDAR currents, in a subset of experiments we applied a GluN2B-containing NMDAR selective inhibitor Ro 25–6891 (Ro, 1 μM; Fischer et al., 1997; Mutel et al., 1998). NMDAR currents resistant to Ro, considered here as currents driven by putative GluN2A-containing NMDARs, were then subtracted from the total NMDAR current. The resulting Ro-sensitive component was considered to be driven by GluN2B-containing NMDARs (Figure 3A). As for initial NMDAR current amplitude (Figure 2), the conclusion that NMDAR function is reduced in the direct pathway of NL1 KO mice was also supported when considering total NMDAR charge transfer (Q) (Figure 3B; WT, 62 ± 5.0 nC, *n* = 19, vs. KO, 41 ± 3.2 nC, *n* = 20, *P* = 0.008). This reduction in NMDAR function was driven mainly by a reduction in GluN2A Q (WT, 35 ± 3.6 nC, *n* = 19, vs. KO, 20 ± 2.5 nC, *n* = 20, *P* = 0.008), but not GluN2B Q (WT, 27 ± 2.4 nC, *n* = 19, vs. KO, 21 ± 2.2 nC, *n* = 20, *P* = 0.089). Again, as for the NMDA/AMPA ratio, DR2 MSNs between WT and NL1 KO littermates showed no significant difference either in total NMDAR Q (Figure 3B; WT, 57 ± 4.9 nC, *n* = 21, vs. KO, 48 ± 4.2 nC, *n* = 22, *P* = 0.121), in Q driven by GluN2A-containing NMDARs (WT, 29 ± 2.4 nC, *n* = 21, vs. KO, 23 ± 2.5 nC, *n* = 22, *P* = 0.295), or in Q driven by GLUN2B-containing NMDARs (WT, 29 ± 2.5 nC, *n* = 21, vs. KO, 23 ± 2.4 nC, *n* = 22, *P* = 0.120), although a “genotype × sex” interaction for GluN2B was present (see Table 1).

GluN2A-containing NMDARs have a higher affinity for glutamate than GluN2B-containing NMDARs (Erreger et al., 2005). In general, their subcellular localization is also different; GluN2A-containing NMDARs are mainly synaptic, whereas GluN2B-containing NMDARs are the predominantly extrasynaptic NMDARs but are also expressed at significant levels at the synapse (Steigerwald et al., 2000; Brickley et al., 2003; Townsend et al., 2003; Kopp et al., 2007). Therefore, the proportion of Glun2A vs. GluN2B-containing NMDARs that are activated can differ depending on the amount of neurotransmitter released. At low neurotransmitter concentrations, synaptic (i.e., mainly GluN2A- but also Glun2B-containing) receptors will be activated, but at strong enough stimulation intensities, glutamate spillover and activation of GluN2B-containing NMDARs may predominate (Kullmann and Asztely, 1998; Carter and Regehr, 2000; Chalifoux and Carter, 2011).

To investigate if this could be happening in our recording conditions, we performed regression analysis comparing the total NMDAR Q among all experiments within the same genotype and pathway to the Q fraction of their corresponding GluN2A or GluN2B components. This analysis indicated no correlation between normalized total NMDAR and the Q fraction driven by either GluN2A- or GluN2B-containing NMDARs, indicating that significant differences in the relative amount of neurotransmitter released among these experiments (partially reflected in the size of the total NMDAR Q) do not exist, and, therefore, do not drive our main observation (Figure 3C, Linear Regression of normalized GluN2A Q vs. total NMDA Q. DR1 WT; slope = 0.20 ± 0.15, *r*^2^ = 0.075, *F*_(1,20)_ = 1.618, *P* = 0.218; DR1 KO; slope = 0.09 ± 0.16, *r*^2^ = 0.017, *F*_(1,19)_ = 0.319, *P* = 0.579; DR2 WT; slope = −0.76 ± 0.12, *r*^2^ = 0.020, *F*_(1,19)_ = 0.394, *P* = 0.538; DR2 KO; slope = −0.10 ± 0.20, *r*^2^ = 0.014, *F*_(1,18)_ = 0.268, *P* = 0.611); Linear Regression of normalized GluN2B Q vs. total NMDA Q. DR1 WT; slope = −0.20 ± 0.15, *r*^2^ = 0.075, *F*_(1,20)_ = 1.638, *P* = 0.215; DR1 KO; slope = −0.09 ± 0.16, *r*^2^ = 0.016, *F*_(1,19)_ = 0.315, *P* = 0.581; DR2 WT; slope = 0.76 ± 0.12, *r*^2^ = 0.020, *F*_(1,19)_ = 0.394, *P* = 0.538; DR2 KO; slope = −0.10 ± 0.20, *r*^2^ = 0.015, *F*_(1,18)_ = 0.268, *P* = 0.611).

### The Frequency of Spontaneous mEPSC Neurotransmission is Reduced in the Indirect Pathway of NL1KO Mice

**Figure 4:**
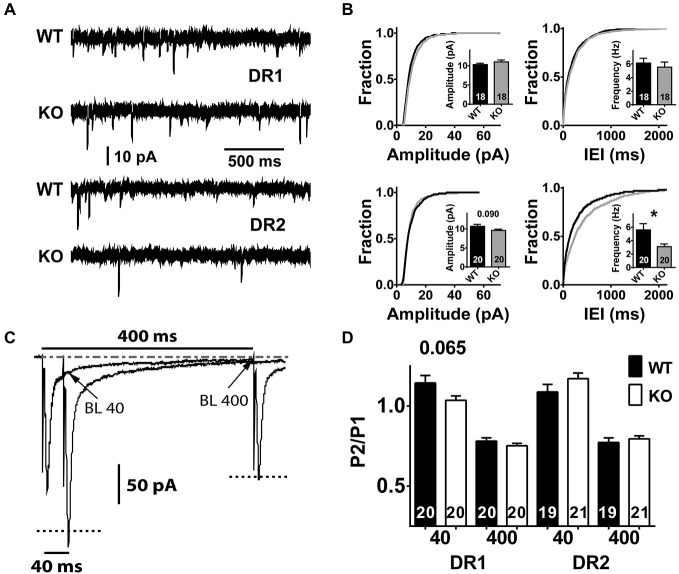
**Characteristics of spontaneous miniature neurotransmission and short-term plasticity in the direct and indirect pathway MSNs of NL1 KO mice. (A)** In the presence of picrotoxin (75–100 μM) and of Tetrodotoxin (0.75–1 μM), spontaneous miniature neurotransmission was recorded. After the membrane patch was broken by suction, the pipette intracellular solution was dialyzed for 8–10 min and recordings were taken for 2–5 min in order to observe at least 200 events per recorded neuron. The upper 2 traces and the lower 2 traces correspond to a 2 s segment recorded in the direct pathway or in the indirect pathway, respectively. The size and number of events look similar between genotypes in DR1 neurons, however, DR2 neurons show fewer events in NL1 KO mice. **(B)** DR2 neurons of NL1 KO mice show increased IEI (bottom right panel) reflected as an approximately 2-fold reduction in the frequency of mini events (inset). Cumulative distributions for amplitudes and for inter-event intervals (IEIs) were built with exactly 200 events per experiment to avoid distribution bias by experiments with higher number of events. **(C)** paired pulse ratio (PPR) average traces at IEIs of 40 ms or 400 ms. Downward deflections for stimulation # 1 for both IEIs are superimposed, therefore, only one event is apparent. The current relaxation after the peak for stimulus # 1 (P1) at 400 ms was used to determine the baseline level (BL) for stimulation # 2 (P2) at both IEI 40 ms and IEI 400 ms (P2–40 and P2–400, respectively). Peak amplitude of P2–40 was calculated by subtracting the amplitude of the BL at the time of the peak of P2 (BL 40) from its absolute amplitude. BL400 was taken as the average current amplitude of a 2 ms window just before P2- 400 was triggered. Similarly, peak amplitude for P2–400 was calculated by subtracting BL400 from the absolute peak value for the corresponding event. The dashed-dotted line at the top corresponds to the steady-state baseline. Dotted lines in both P2s correspond to their recalculated amplitudes after baseline subtraction. **(D)** No significant changes in PPR were identified between genotypes in either pathway, though a trend is apparent in DR1 neurons at IEI of 40 ms. *P < 0.05.

*In vitro* and *in vivo* experimental procedures that affect NL1 expression levels can impact spontaneous miniature excitatory neurotransmission (Prange et al., 2004; Nam and Chen, 2005; Chen et al., 2010; Mondin et al., 2011; Burton et al., 2012; Kwon et al., 2012). Furthermore, studies in cortex and hippocampus have shown that NL1 levels modulate synaptic vesicle accumulation and neurotransmission (Wittenmayer et al., 2009; Stan et al., 2010), and synaptic release probability (Futai et al., 2007). Thus, we reasoned that constitutive deletion of NL1 may affect spontaneous miniature excitatory neurotransmission in the striatum. To investigate this, we compared the properties of miniature excitatory synaptic transmission in both DR1 and DR2 MSN pathways between NL1 KO and WT mice. NL1 KO mice showed no difference in mEPSC neurotransmission in the direct pathway (WT amplitude, 10.3 ± 0.34 pA, *n* = 18 vs. KO amplitude, 11 ± 0.48 pA, *n* = 18, *P* = 0.850; WT frequency, 6.1 ± 0.70 Hz, *n* = 18 vs. KO frequency, 5.5 ± 0.76 Hz, *n* = 20, *P* = 0.755). The lack of mEPSC amplitude difference is consistent with our conclusion that the direct pathway alterations in NMDA/AMPA ratio are mediated by altered NMDAR-mediated currents as no change in the largely AMPAR-mediated mEPSC was identified. However, mEPSC frequency was reduced in the indirect pathway (Figures 4A,B; WT frequency, 5.6 ± 0.92 Hz, *n* = 20; KO frequency, 3.1 ± 0.40 Hz, *n* = 20, *P* = 0.023). A small change was also observed in the amplitude in the indirect pathway, but it did not reach statistical significance (WT amplitude, 10.7 ± 0.53 pA, *n* = 20 vs. KO amplitude, 9.6 ± 0.33 pA, *n* = 20, *P* = 0.090).

### Marginal Differences in Paired Pulse Facilitation in NL1 KO Mice

A reduction in miniature frequency may be related to a decrease in release probability of synaptic vesicles. PPR is frequently used as an initial approach to investigate changes in the probability of neurotransmitter release (Salin et al., 1996; Kaplan et al., 2003). PPR is studied by pairing two stimuli of the same inputs with specific, brief inter-stimulus intervals (inter-event interval, IEI). Our unpublished data and those reported by other groups (Akopian and Walsh, 2007; Ding et al., 2008) indicate that stimulation of glutamatergic afferents to the striatum at short IEIs (i.e., 30–70 ms) induce a mild facilitation whereas depression is recorded at IEIs above 150 ms. Within the same PPR protocol, here we used IEI 400 ms (in odd sweeps) and IEI 40 ms (in even sweeps) because they elicit the maximum levels of depression and of facilitation, respectively.

To determine if PP facilitation (PPF) or PP depression (PPD) existed within a group, we first compared currents elicited by pulse 1 and 2 for each IEI, followed by comparison of PPRs between groups. Both, DR1 and DR2 pathways showed significant levels of depression at the IEI of 400 ms (DR1 WT-P1, −197 ± 6.9 pA, vs. DR1 WT-P2, −153 ± 6.3 pA, *n* = 20, *P* < 0.001; DR1 KO-P1, −222 ± 7.1 pA vs. DR1 KO P2, −166 ± 4.8, *P* < 0.001, *n* = 20; DR2 WT-P1, −210 ± 6.1 pA vs. DR2 WT-P2, −161 ± 5.8 pA, *n* = 20, *P* < 0.001; DR2 KO-P1, −196 ± 17.6 pA vs. DR2 K0-P2, −153 ± 13.3, *n* = 20, *P* < 0.001). When considering the PPR at this IEI, no difference between genotypes was observed (Figures 4C,D; DR1 WT, −0.78 ± 0.02 pA, *n* = 20 vs. DR1 KO, −0.75 ± 0.01 pA, *n* = 20, *P* = 0.592; DR2 WT, −0.77 ± 0.03 pA, *n* = 19, vs. DR2 KO, −0.80 ± 0.02 pA, *n* = 21, *P* = 0.485). Again, at the short IEI of 40 ms, both genotypes showed similar results (i.e., facilitation) in the DR2 pathway (WT P1, −207 ± 5.7 pA vs. WT P2, −225 ± 10.5 pA, *n* = 19, *P* = 0.011; KO P1, −224 ± 7.6 pA vs. KO P2 −231 ± 8.8, *n* = 20, *P* < 0.001), with no difference in PPR between genotypes (WT, −1.09 ± 0.05 pA, *n* = 20, vs. KO, 1.17 ± 0.04 pA, *n* = 21, *P* = 0.157). On the contrary, in the DR1 pathway, WT mice showed significant facilitation at 40 ms IEI, but not NL1 KO (WT P1, −197 ± 6.9 pA vs. WT P2 −223 ± 10.5 pA, *P* = 0.002, *n* = 20; KO P1, −224 ± 7.6 pA vs. KO P2, −231 ± 8.8, *n* = 20, *P* = 0.355). However, when PPRs were compared between WT and KO at this short interval, no significance (although a trend) was reached in the DR1 pathway (WT, −1.14 ± 0.05 pA, *n* = 20, vs. DR1 KO, 1.04 ± 0.03 pA, *n* = 21, *P* <= 0.065). These findings suggest no alterations in presynaptic release probability to explain the altered mEPSC frequency in the indirect pathway.

**Figure 5:**
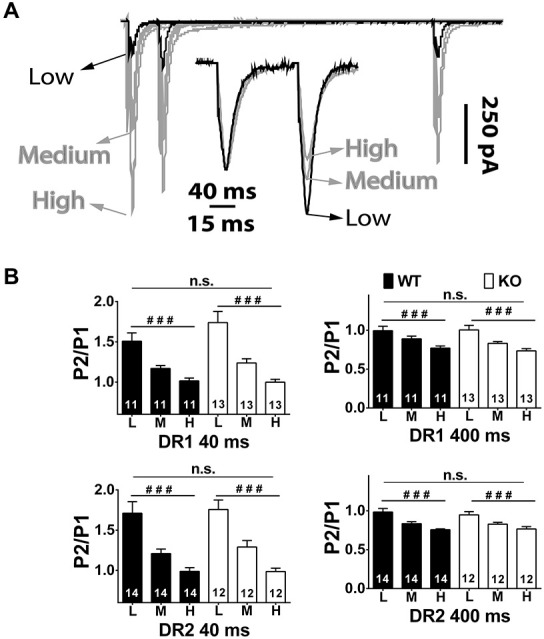
**Short-term plasticity in the direct and indirect pathway MSNs of NL1 KO mice at various divalent concentrations. (A)** Main traces, similar conditions as in Figure 1 but at low (0.6 mM), medium (1.5 mM) and high (2.9 mM) [Ca^2+^]*_e_*. To maintain total divalent concentration constant, Mg^2+^ concentrations were (in mM); 2.4, 1.5 and 0.1, respectively. The peak amplitude of P1 as well as PP Depression (PPD) at IEI 400 ms showed a strong positive correlation to [Ca^2+^]*_e_*. Instead, after normalizing traces at IEI 40 ms (inset), it is evident that PP Facilitation (PPF) showed an inverted relation to [Ca^2+^]*_e_*, that is, the larger the [Ca^2+^]*_e_*, the smaller the PPF. **(B)** As expected, repeated measures ANOVA showed a strong effect of [Ca^2+^]*_e_*, on PPF and on PPD on both genotypes, but no significant changes in PPR between genotypes in either IEI and pathway were found. ^###^*P* < 0.001.

The PPR experiments described above were conducted in regular aCSF, where a marginal difference if any between genotypes was found in the direct pathway. To further the chances of unmasking release probability differences between genotypes, additional paired experiments at three concentrations of Ca^2+^ ([Ca^2+^]*_e_*) were performed (Figures 5A,B). The [Ca^2+^]*_e_* used were 0.6, 1.5 and 2.9 mM. Because regular aCSF contains 2 mM Ca^2+^ and 1mM Mg^2+^, [Mg^2+^]*_e_* was varied to keep the total divalent concentration constant at 3 mM. Also, given that significant NMDAR currents are observed at the highest [Ca^2+^]*_e_* (2.9 mM), where [Mg^2+^]*_e_* is only 0.1 mM, (2*R*)-amino-5-phosphonovaleric acid; (2*R*)-amino-5-phosphonopentanoate (APV) 75–100 μM was included in the bathing solutions. Importantly, as shown in previous reports, APV did not affect PPF nor PPD by itself, suggesting that NMDARs are not involved in these phenomena (not shown). As expected, in the long IEI interval (400 ms), PPD increased with [Ca^2+^]*_e_* (DR1 WT PPR low, 0.99 ± 0.042; medium, 0.89 ± 0.026; high, 0.77 ± 0.028; *n* = 11, *P* < 0.001. DR1 KO PPR low, 1.00 ± 0.058; medium, 0.83 ± 0.023; high, 0.73 ± 0.029; *n* = 13, *P* < 0.001. DR2 WT PPR low, 0.98 ± 0.046; medium, 0.83 ± 0.025; high, 0.76 ± 0.012; *n* = 14, *P* < 0.001. DR2 KO PPR low, 0.95 ± 0.041; medium, 0.83 ± 0.024; high, 0.77 ± 0.030; *n* = 12, *P* < 0.001), but the repeated measures ANOVA yielded no significant difference between genotypes (*P* = 0.691, for DR1 pathway; and, *P* = 0.776, for DR2 pathway). At IEI 40 ms, PPF was inversely correlated with [Ca^2+^]*_e_* (DR1 WT PPR low, 1.51 ± 0.104; medium, 1.17 ± 0.037; high, 1.02 ± 0.036; *n* = 11, *P* < 0.001. DR1 KO PPR low, 1.74 ± 0.138; medium, 1.24 ± 0.052; high 1.00 ± 0.036; *n* = 13, *P* < 0.001. DR2 WT PPR low, 1.71 ± 0.144; medium, 1.21 ± 0.057; high, 0.99 ± 0.046; *n* = 14, *P* < 0.001. DR2 KO PPR low, 1.84 ± 0.119; medium, 1.29 ± 0.088; high 0.99 ± 0.042; *n* = 12, *P* < 0.001.), but, again, no difference was found between genotypes (*P* = 0.302, for DR1 pathway; and, *P* = 0.773, for DR2 pathway). Overall, the lack of significant alterations in PPR across genotypes in both direct and indirect pathways suggests that the decrease observed in the indirect (D2) MSN mEPSC frequency is not likely due to alterations in probability of release.

## Discussion

Our findings suggest a decrease in NMDAR-mediated synaptic transmission in NL1 KO mice that is selective for DR1 (direct pathway) MSNs. More specifically, the reduction of NMDAR currents in DR1 neurons is mainly due to decreased currents driven by NMDARs containing the GluN2A subunit, although a trend in reduced currents from GluN2B containing NMDARs is also present. These findings confirm and extend our previous report on an overall decrease in NMDA/AMPA ratio in the total pool of striatal MSNs (Blundell et al., 2010). Additionally, we also show that mEPSC frequency is reduced in the indirect pathway with no change in PPR.

While it is clear that NMDAR-mediated currents are decreased in the direct pathway of NL1 KO mice and the mechanism of this decrease is due to alterations in GluN2A containing receptors, the molecular mechanisms involved in the reduction of NMDAR/GluN2A currents in the direct pathway in NL1 KO mice remain uncertain. That said, several lines of evidence suggest that NL1 modulates NMDAR function at excitatory synapses. First, NL1 is preferentially, if not uniquely, localized to excitatory synapses (Song et al., 1999; Chubykin et al., 2007; Dahlhaus et al., 2010). Second, NL1 has been shown to have indirect (through PSD-95; Irie et al., 1997; Prange et al., 2004; Gerrow et al., 2006; Barrow et al., 2009) and, direct (through an extracellular domain) physical interaction with NMDARs (Budreck et al., 2013). These interactions have been shown to be important to induce and stabilize clusters of NMDARs at the synapse, both during development and during adulthood, and may favor the lateral movement of NMDARs from extrasynaptic to synaptic locations upon use-dependent blockage of synaptic NMDARs (Tovar and Westbrook, 2002; Budreck et al., 2013). Third, manipulations of NL1 expression levels do indeed demonstrate a strong positive correlation with the amplitude of NMDAR currents and with the NMDA/AMPA ratio in other brain regions and neurons (Chubykin et al., 2007; Futai et al., 2007; Kim et al., 2008b; Jung et al., 2010; Soler-Llavina et al., 2011; Kwon et al., 2012; Budreck et al., 2013). These data strongly suggest that the levels of NMDAR currents are in part controlled by NL1 without a concomitant alteration of AMPAR currents. However, when NL1 is overexpressed, changes in NMDAR currents do not always correlate with similar changes in NMDA/AMPA ratio because AMPAR currents are also increased (Futai et al., 2007; Shipman et al., 2011; Schnell et al., 2012; Shipman and Nicoll, 2012). Also, in some of these reports, the changes in both AMPA and NMDAR-mediated currents were in line with changes in the number of synapses (Shipman et al., 2011; Schnell et al., 2012; Shipman and Nicoll, 2012), suggesting that the changes in synaptic currents were in part a reflection of the changes in synapse number.

In our study, we identified decreased NMDAR currents only in D1 MSNs of the direct pathway but no alterations in mEPSC frequency in the direct pathway to suggest altered synapse number, and no change in mEPSC amplitude to suggest alterations in AMPAR-mediated synaptic responses. Likewise, we identified no alterations in D1 MSN input/output curves to suggest decreased synapse number. This is in line with our previous report regarding synapse numbers in hippocampus (Blundell et al., 2010) and from another group in cortex (Kwon et al., 2012), where constitutive loss of NL1 did not induce a decrease in synapse number. Thus, it appears that NL1 deletion can selectively alter NMDAR-mediated currents in MSNs of the direct pathway. This effect is largely mediated by alterations in NMDARs containing GluN2A subunits. The mechanism of this effect as well as its selectivity for direct pathway MSNs and for GluN2A-containing NMDARs is of great interest for future studies.

There are numerous potential explanations for the synapse specificity of the effects of NL1 KO. In NL1 KO mice, the effect of constitutively ablating NL1 expression could differ among glutamatergic synapses onto the direct pathway vs. glutamatergic synapses onto the indirect pathway, perhaps due to differences among these synapses in the expression of specific presynaptic neurexins, of leucine-rich repeat transmembrane protein isoforms (LRRTMs), or of other neuroligins that compete with NL1 for neurexin binding (Ko et al., 2009a; Linhoff et al., 2009; Shipman et al., 2011; Soler-Llavina et al., 2011). Furthermore, differential expression of NL1 and its partners/competitors among different synapses could also explain the selective effects that manipulating NL1 expression levels have on plastic properties observed in glutamatergic inputs to the amygdala from cortex vs. those from the thalamus (Jung et al., 2010), as well as the differences in levels of glutamatergic currents and on plastic properties between CA1 and dentate gyrus neurons in the adult hippocampus (Shipman and Nicoll, 2012). In line with the idea that expression levels of some synaptic proteins vary between direct and indirect pathways, differential mRNA expression levels between striatonigral MSNs and striatopallidal MSNs has been shown for Nxn1 (Lobo et al., 2006) as well as for NL3 (Rothwell et al., 2014).

Striatal synaptic NMDAR currents and/or the NMDA/AMPA ratio are also affected in other animal models of ASD and obsessive compulsive disorder (OCD; Welch et al., 2007; Peça et al., 2011; Kouser et al., 2013). This reduction in NMDAR currents can correlate with a reduction in NMDAR subunit protein levels in total brain extracts (Peça et al., 2011). Similarly, a generalized increase in the levels of GluN1 or GluN2 subunits is induced upon NL1 overexpression (Budreck et al., 2013; Hoy et al., 2013), supporting the idea that the size of synaptic NMDAR currents can correlate with a generalized alteration of NMDAR expression levels. In stark contrast, no reduction in the levels of NMDAR subunits due to constitutive loss of NL1 *in vivo* has been demonstrated to our knowledge. For example, in spite of a reduction of NMDAR currents in NL1 KO mice (Blundell et al., 2010; Budreck et al., 2013), no changes in levels of these subunits were demonstrated either in total brain homogenates or in biotinylated surface proteins. Together, this evidence suggests that mechanisms other than protein expression levels are involved in the reduction of NMDAR currents in NL1 KO mice. Budreck et al. (2013) indicate that NL1 and NMDARs directly interact through extracellular domains. This interaction seems to be important for the recruitment of extrasynaptic NMDARs into the synapse, stabilizing clusters of synaptic NMDARs without significantly affecting total NMDAR expression levels or synapse numbers (Budreck et al., 2013). Additionally, constitutive loss of NL1 may induce other type of posttranslational modifications that impact NMDAR channel function that could contribute to reduce NMDAR currents without affecting NMDAR expression levels.

We and others have demonstrated altered mEPSC synaptic transmission in response to experimental manipulations that modify the expression levels of NL1. In general, NL1 overexpression increases mEPSC frequency, whereas NL1 ablation, knockdown, or expression of dominant-negative NL1, decreases it (Prange et al., 2004; Nam and Chen, 2005; Chen et al., 2010; Mondin et al., 2011; Burton et al., 2012; Kwon et al., 2012; Schnell et al., 2012). Even though NL1 is a postsynaptic protein, it has been shown to modulate synaptic vesicle clustering through direct or indirect interactions with presynaptic proteins. Indeed, overexpression of NL1 increases the number of vesicles at the synapse (Dahlhaus et al., 2010), an effect that depends on the presence of functional scaffolding proteins S-SCAM, on the postsynaptic side, and N-cadherin, presynaptically (Stan et al., 2010). In addition, synaptic vesicles can also be modulated by the association of NL1 with β-neurexin (Dean et al., 2003). In this regard, we previously reported that the NL1 KO brain shows a significant reduction in several presynaptic proteins including Nxn-1 and mammalian uncoordinated-18 (Munc18; Blundell et al., 2010). These alterations alone could be affecting neurotransmitter release because Munc18 is essential for synaptic vesicle priming (Deák et al., 2009), whereas neurexins, besides modulating synaptic clustering, they can also link presynaptic Ca^2+^ channels to the synaptic vesicle release machinery (Missler et al., 2003; Kattenstroth et al., 2004; Zhang et al., 2005). Of related interest are the findings of Lobo et al describing differential mRNA expression between direct and indirect pathway MSNs, including a 2-fold difference in neurexin-1 expression (Lobo et al., 2006). However, NL1 KO mice showed no difference in PPR, even at various Ca^2+^ concentrations, making the latter possibility of low likelihood. Importantly, multiple lines of evidence indicate that evoked and spontaneous neurotransmission may differ in the modulation of synaptic vesicle release properties (Smith et al., 2012) which may even utilize different pools of synaptic vesicles (Kavalali, 2015). This varies among brain regions and synapse types and could be due to local synapse-specific expression of synaptic proteins that modulate both types of neurotransmission. Likewise, differences in the baggage of synaptic proteins that comprise local release machineries could be involved in the distinct effects between striatal pathways reported here. If this were the case, the constitutive absence of NL1 could lead mainly to postsynaptic alterations in the direct pathway vs. mainly presynaptic alterations in the indirect pathway. Our present results leave open this interesting and complex line of inquiry for future studies.

Similar to several other mouse models of ASD or of OCD (Greer and Capecchi, 2002; Welch et al., 2007; Etherton et al., 2009; Shmelkov et al., 2010; Peça et al., 2011), NL1-null mice show an ~2-fold increase in repetitive grooming (Blundell et al., 2010). We do not presently know what the specific impact of the changes in striatal neurotransmission reported here may have on grooming behavior of NL1 KO mice, however, some testable hypotheses may be drawn from the literature. Given that activation of the direct pathway using DR1 agonists increased grooming behavior in rodents (Van Wimersma Greidanus et al., 1989; Berridge and Aldridge, 2000; Taylor et al., 2010), whereas facilitation of the indirect pathway by pretreatment with DR2 antagonists prevented the effect of DR1 agonists on grooming (Molloy and Waddington, 1987; Taylor et al., 2010), the emerging model is that dopamine facilitates the activation of the direct pathway while, at the same time, it dampens the inhibitory effects of the indirect pathway on motor program completion. Importantly, antagonism of Glun2A containing-NMDARs, favors dopaminergic release (Schotanus and Chergui, 2008) and, therefore, could have a stimulating impact on grooming. This is in agreement with the rescue of grooming behavior that systemic injections of D-cycloserine have in NL1 KO mice. However, it will remain to be determined in future studies if reductions in NMDAR currents are intertwined with the dopaminergic modulation of grooming in this mouse.

In summary, we have demonstrated reduced NMDAR currents selectively in the direct pathway MSNs of dorsal striatum in NL1 KO mice. This is due to a selective decrease of GluN2A-containing NMDAR currents at excitatory synapses onto direct pathway MSNs. Furthermore, action potential-independent spontaneous neurotransmission is selectively reduced in the indirect pathway of NL1 KO mice. These findings correlate altered striatal synaptic function to the increased grooming phenotype of NL1 KO mice. Indeed they lead to testable hypotheses of how altered striatal synaptic function may result in increased repetitive grooming.

## Conflict of Interest Statement

The authors declare that the research was conducted in the absence of any commercial or financial relationships that could be construed as a potential conflict of interest.
